# Regenesis: Repair and regeneration reinvented in stem cell therapeutics

**DOI:** 10.1016/j.stemcr.2026.102825

**Published:** 2026-02-26

**Authors:** Hitesh Chopra, Yuanyuan Han

**Affiliations:** 1 Department of Periodontics and Oral Medicine, School of Dentistry, University of Michigan, Ann Arbor, MI 48109, USA

## Abstract

Despite rapid advancements in stem cell-based therapies, the repair-regeneration dichotomy often fails to capture most stem cell-mediated outcomes. We propose “regenesis**”** as an integrative framework describing hybrid tissue outcomes. This commentary highlights the conceptual gap and aims to stimulate discussion on updating terminology in stem cell therapeutics.

## Graphical abstract

**Figure:**
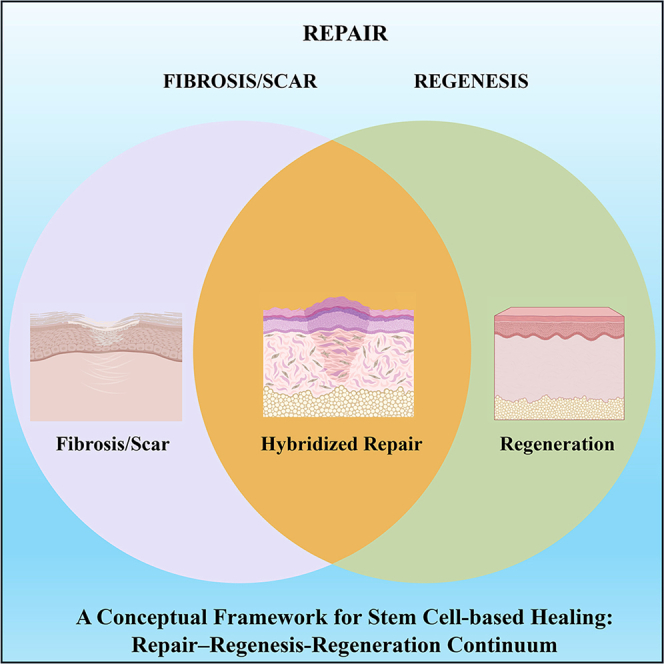

## Introduction

**Figure 1:**
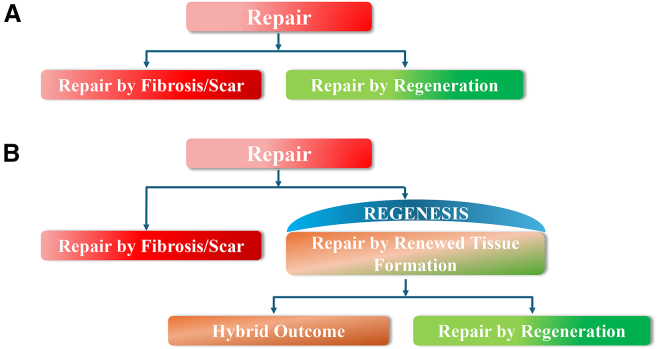
Classical and refined frameworks of tissue repair in stem cell-based healing (A) Classical wound-healing framework, in which tissue repair is traditionally categorized as repair by fibrosis (scar-dominant healing) or repair by regeneration (complete structural and functional restoration). (B) Refined framework incorporating regenesis within the classical repair paradigm. Regenesis denotes renewed tissue formation following injury and encompasses a spectrum of outcomes, including hybrid healing responses characterized by partial tissue renewal with concurrent fibrosis, as well as regeneration, defined by complete structural and functional restoration without fibrosis. Most stem cell-based therapies produce regenesis rather than pure fibrosis or complete regeneration.

Over the past two decades, stem cell research has expanded at an extraordinary pace, reshaping what medicine can realistically hope to repair, regenerate, or replace. Yet despite these rapid technological and conceptual advances, the vocabulary used to describe tissue outcomes remains anchored in outdated dichotomies. Repair is classically defined as healing through fibrotic deposition and scar formation (Gurtner et al., 2008; Robbins, 1957), whereas regeneration refers to the replacement of damaged tissues with newly formed, fully functional tissues (Carlson, 2011; Gurtner et al., 2008) (Figure 1A). Functionally, repair may or may not restore normal organ performance, depending on the tissue context. For example, epithelial repair can fully re-establish barrier function, whereas the fibrotic scar formed after myocardial infarction permanently compromises cardiac physiology. In contrast, regeneration by definition culminates in the complete structural and functional restoration.

Classic model organisms such as planarians and certain parasitic flatworms, including tapeworms, exhibit near-limitless regenerative capacity, making them indispensable for uncovering the cellular and molecular mechanisms that distinguish true regeneration from repair. Their biological capabilities, however, are fundamentally distinct from—and far exceed—the regenerative potential achievable in current stem cell-based therapeutics.

In reality, stem cell-based interventions rarely yield pure regeneration or simple repair. Instead, they elicit a spectrum of biological responses combining paracrine signaling, extracellular matrix remodeling, immune modulation, and tissue restoration (Baraniak and McDevitt, 2010; Gnecchi et al., 2008; Hodgkinson et al., 2016). Forcing these complex outcomes into a strict repair-regeneration dichotomy oversimplifies and sometimes mischaracterizes the processes we are measuring. Therefore, within the limitations of current scientific knowledge, it is inaccurate to label clinical approaches as “regenerative orthopedics” or “regenerative endodontics” in medicine and dentistry, respectively.

This conceptual mismatch is not just semantic. It influences how results are interpreted, how treatments are marketed, and how expectations are shaped for patients, clinicians, and regulatory agencies. As stem cell science advances, often through incremental and transformative discoveries, a more precise conceptual framework is needed. We propose that adopting the term “regenesis” in stem cell research may stimulate scientific debate, encourage critical thinking, and promote a more accurate recognition of the hybrid biological processes that dominate stem cell-mediated healing. In doing so, it provides a clearer and more constructive framework for the broader scientific community.

## Why repair and regeneration are No longer adequate in the context of stem cell therapies?

The classical dichotomy of repair versus regeneration arose from early wound-healing biology, where tissues were thought to follow one of two trajectories: imperfect restoration with scar (repair) or complete structural and functional reconstitution (regeneration). While this framework worked for describing spontaneous healing processes, it has become increasingly insufficient to explain how modern stem cell-based therapies influence tissue outcomes. The mechanistic diversity uncovered over the past two decades does not fit cleanly into either category. Three major reasons illustrate why the binary language falls short.

### Stem cells rarely act by direct replacement

The expectations for stem cell therapy are that the transplanted cells would engraft, differentiate, and replace lost tissue—fulfilling a textbook definition of regeneration. However, cumulative evidence across multiple organs shows that direct replacement is the exception, not the rule. In most tissues, such as bone, myocardium, or dental pulp, transplanted stem cells exert their benefits predominantly through paracrine effects: growth factors, cytokines, and extracellular vesicles that stimulate host repair mechanisms (Brizuela et al., 2022; Caplan and Correa, 2011; Granero-Molto et al., 2009; Hodgkinson et al., 2016; Weryha and Leclere, 1995). These processes do not fit neatly within the traditional repair or regeneration categories. Instead, they involve a blend of partial restoration, partial scarring, and significant biological correction of dysfunctional pathways.

### Many tissues heal through mixed mechanisms

Current evidence shows that many tissues respond to stem cell-based interventions through mixed processes rather than through pure repair or pure regeneration. Some of the examples include the following.

(1) Bone defects treated with mesenchymal stem cells (MSCs) exhibit areas of new mineralized bone along with regions of fibrous repair at margins, creating a hybrid tissue architecture (Granero-Molto et al., 2009).
(2) Cardiac injuries treated with cell therapy rarely achieve full replacement of lost cardiomyocytes. Instead, they promote remodeling of fibrotic tissue, enhance vascular support, and improve cardiac function (Madonna et al., 2016).
(3) Dental pulp regeneration procedures often produce tissue that resembles pulp in structure and function but does not fully recapitulate all cellular elements or architectural features of native pulp tissue (Kontakiotis et al., 2015).

These outcomes demonstrate that stem cell therapies tend to produce partial restoration combined with tissue remodeling rather than pure repair or pure regeneration.

### The binary language stifles conceptual clarity

Disciplines such as “regenerative medicine,” “regenerative orthopedics,” or “regenerative endodontics” often use the term “regeneration” aspirationally, referring more to therapeutic intent than biological outcome. This creates an unintended gap between what is claimed and what is biologically possible, which can elevate expectations and ultimately foster misalignment among researchers, clinicians, patients, and regulatory agencies.

Replacing the oversimplified language with more accurate concepts—such as tissue modulation, functional restoration, or biological reprogramming of healing—would better reflect contemporary science and promote clearer communication.

## Etymology of the term regenesis

The term regenesis is derived from classical linguistic roots: the prefix re- (Latin), meaning “again,” and genesis (Greek γένεσις), meaning “origin,” “formation,” or “creation.” In biological contexts, genesis refers to the process of tissue formation rather than the nature or completeness of the resulting structure, as reflected in widely accepted terms such as angiogenesis, osteogenesis, and neurogenesis. Accordingly, regenesis denotes renewed tissue formation following injury, without presupposing complete restoration of native tissue architecture.

This distinction is central to contemporary stem cell therapeutics. While regeneration represents an idealized biological outcome, cumulative evidence demonstrates that in most stem cell-based interventions, repair and regeneration occur concurrently or sequentially, resulting in partial structural restoration alongside functional improvement driven by both cellular and paracrine mechanisms.

Within this framework, regenesis serves as an umbrella concept encompassing a spectrum of tissue outcomes that involve renewed tissue formation (Figure 1B). These outcomes include hybrid healing responses, characterized by partial tissue renewal combined with scar formation, as well as true regeneration, defined by complete structural and functional restoration. Many stem cell-mediated outcomes occupy intermediate positions within this continuum, combining elements of both fibrotic repair and regenerative renewal. Importantly, all regeneration represents a form of regenesis, but not all regenesis culminates in true regeneration. In contrast, purely fibrotic repair in which healing occurs through scar formation, without meaningful tissue renewal, lies outside the regenesis spectrum.

To describe interventions that promote such outcomes, we use the adjective regenic, denoting therapies and approaches that induce regenesis without presuming complete architectural or functional restoration, such as regenic orthopedics or regenic endodontics. This terminology acknowledges biological reality, preserves the strict definition of regeneration, and avoids overextension of aspirational language. By adopting regenesis as a conceptual framework, we aim to provide a more precise and biologically grounded alternative to the traditional repair-regeneration dichotomy for interpreting stem cell-mediated healing and to facilitate clearer communication across experimental, clinical, and regulatory contexts.

## Why does the concept of regenesis add value?

(1) It reflects biological reality rather than idealized goals.
(2) It clarifies the mechanism, since paracrine-driven tissue improvement is neither fibrotic repair/scarring nor complete regeneration.
(3) It encourages honest and transparent reporting of therapeutic outcomes without overextending claims of regeneration.
(4) It supports more accurate communication among researchers, clinicians, and regulatory agencies.
(5) It reframes the field toward incremental and measurable improvement rather than the expectation of perfect regeneration or structural and functional restoration.

## Why the field needs this concept now?

Stem cell science, although still exploratory in many aspects, is rapidly entering clinical application, commercialization, and regulatory scrutiny. As the field evolves, precision of scientific language becomes a responsibility rather than a preference. Therefore, regenesis serves as the following.

(1) A conceptual anchor for interpreting hybrid healing outcomes.
(2) A corrective to the overuse of the term “regeneration.”
(3) A framework to stimulate debate and refine classification systems.
(4) A step toward aligning mechanistic understanding with clinical language.

## Conclusion: A call for conceptual precision

The era of stem cell-based medicine demands vocabulary that reflects biological truth rather than simplified categories. Repair and regeneration, although historically important, no longer capture the layered complexity of tissue responses that arise through stem cell-driven healing. The concept of regenesis offers a precise, transparent, and constructive framework for describing these hybrid and intermediate outcomes.

Adopting this term will not solve every conceptual problem, but it can start the conversation the field urgently needs—one that challenges old assumptions, sharpens scientific language, preserves the strict definition of regeneration, and enables researchers to communicate more clearly about the remarkable, but rarely binary outcomes of stem cell therapy.

## Acknowledgments

The authors acknowledge the researchers and specialists from their field for their contributions in stem cell research/therapy that served as a foundation for developing this concept.

## Author contributions

H.C.: conceptualization, writing – original draft, and visualization; Y.H. and H.C.: writing – review and editing. Both authors approved the final commentary.

## Declaration of interests

The authors declare no competing interests.
